# A158 APPLYING MACHINE LEARNING FOR PREDICTING TREATMENT RESPONSE TO VEDOLIZUMAB IN PEDIATRIC IBD BY SERUM METABOLOMICS

**DOI:** 10.1093/jcag/gwae059.158

**Published:** 2025-02-10

**Authors:** R G Suarez Suarez, O Bar Or, G Focht, Z Shavit, E Orlanski-Meyer, E Broide, D Urlep, J Hyams, J Levine, J Rosh, D Turner, E Wine

**Affiliations:** Paediatrics, University of Alberta, Edmonton, AB, Canada; Paediatrics, University of Alberta, Edmonton, AB, Canada; Juliet Keidan Institute of Pediatric Gastroenterology, The Hebrew University of Jerusalem, Israel; Juliet Keidan Institute of Pediatric Gastroenterology, The Hebrew University of Jerusalem, Israel; Juliet Keidan Institute of Pediatric Gastroenterology, The Hebrew University of Jerusalem, Israel; Shamir Medical Center Assaf Harofeh, Tzrifin, Central, Israel; University Children’s Hospital of the University Medical Centre Ljubljana, Ljubljana, Slovenia; Connecticut Children’s Medical Center, Hartford, CT; NYU Langone Health, New York, NY; Atlantic Children’s Medical Center, New Jersey, NY; Juliet Keidan Institute of Pediatric Gastroenterology, The Hebrew University of Jerusalem, Israel; Paediatrics, University of Alberta, Edmonton, AB, Canada

## Abstract

**Background:**

Vedolizumab (VDZ) is effective to induce remission in children with Crohn disease (CD) and ulcerative colitis (UC), but effectiveness varies.

Metabolites produced by interactions between intestinal microbiota and host metabolic processes can be useful to identify metabolome signatures that may preferentially favor response to a specific therapeutic class. Therefore, metabolomic studies can potentially inform precision medicine in Inflammatory Bowel Diseases (IBD).

**Aims:**

This study aimed to apply machine learning to assess metabolites as potential predictors for forecasting the response to VDZ treatment.

**Methods:**

VedoKids is a multicenter, prospective, observational cohort study, designed to report the effectiveness and safety of VDZ. Children aged 0-18 years, diagnosed with IBD, who initiated VDZ treatment at any stage of their condition, were subjected to comprehensive assessments at the onset and subsequently at 2, 6, 14, 30, 54 weeks and thereafter. Detailed demographic, clinical, and safety information was meticulously recorded in a prospective manner throughout the study period. Metabolomic profiling was conducted in serum at three time points: baseline, 14 weeks, and 30 weeks. Metabolites were identified using a quantitative metabolomics approach utilizing DI/LC-MS/MS technology for the analysis of serum samples.

We constructed a learning algorithm to predict treatment response to identify the most relevant serum metabolites subset. The algorithm consisted of a Random Fores (RF) model and maximum relevance minimum redundancy (mRMR) feature selection. Response was defined as decrease of ≥20 points in PUCAI or >20 points in wPCDAI and clinical remission as PUCAI<10 or wPCDAI<12.5.

**Results:**

We were able to train different RF models using clinical, pre-treatment, and serum metabolite data. Results for the experiment performed with CD patients at week 14 shows an AUC = 0.95 with N-Acetyl-Aspartic acid, Fumaric acid, and Glutamine scoring among the features with highest predictive importance. Results for the experiment performed with UC patients at week 30 shows an AUC = 0.81 with Isoleucine, Malic acid, and N-Acetyl-Glutamic acid scoring among the features with highest predictive importance.

**Conclusions:**

Our results suggest that it is possible to produce predictor capable of predicting response to VDZ treatment using clinical and metabolome data in children with IBD. Importantly, some of the identified metabolites have been previously associated with IBD pathophysiology.

**Funding Agencies:**

CIHR

